# Role and mechanism of miRNA in cardiac microvascular endothelial cells in cardiovascular diseases

**DOI:** 10.3389/fcvm.2024.1356152

**Published:** 2024-03-13

**Authors:** Junyuan Yan, Xinqin Zhong, Yucui Zhao, Xiaoying Wang

**Affiliations:** ^1^ Ministry of Education Key Laboratory of Pharmacology of Traditional Chinese Medical Formulae, Tianjin University of Traditional Chinese Medicine, Tianjin, China; ^2^ School of Chinese Materia Medica, Tianjin University of Traditional Chinese Medicine, Tianjin, China; ^3^ State Key Laboratory of Component-Based Chinese Medicine, Tianjin University of Traditional Chinese Medicine, Tianjin, China

**Keywords:** cardiac microvascular endothelial cell, miRNA, coronary artery disease, microvascular dysfunction, diabetes, crosstalk

## Abstract

The occurrence and development of myocardial dysfunction are associated with damage in the cardiac microvascular endothelial cells (CMECs), which can regulate nutrient exchange and oxy-gen-carbon cycling to protect cardiomyocytes. Interventions targeting microRNAs (miRNAs) can effectively mitigate CMEC injury and thus improve cardiovascular diseases. MiRNAs are a class of noncoding single-strand RNA molecules typically 21–23 nucleotides in length that are encoded by endogenous genes. They are critical regulators of organism development, cell differentiation, metabolism, and apoptosis. Current clinical trials on miRNA drugs indicate that patient-specific miRNA levels are now being used as one of the criteria for predicting heart disease. However, the cellular process of various miRNAs in CMECs in cardiovascular diseases has not been fully elucidated. These mechanisms are a field that immediately requires further investigation. Accordingly, this review summarizes the roles and mechanisms of various miRNAs in CMECs in cardiovascular disease and includes the process of CMEC crosstalk between miRNAs and other cell types in the heart. Our study serves as a theoretical basis for the formal introduction of miRNA use into the treatment of cardiovascular diseases in the future.

## 1 Introduction

Cardiovascular disease (CVD) now accounts for one-third of all fatalities globally, and it has recently emerged as one of the most significant variables affecting people's health (1). Coronary artery disease (CAD) is the most common type of CVDs and is caused by atherosclerotic blockage of the coronary arteries (2). Its clinical manifestations are usually myocardial infarction (MI) and ischemic cardiomyopathy (3). CAD is becoming more common every year in developed and developing countries, which is seriously endangering people's lives (3, 4). Inflammation, oxidative stress, autophagy, and apoptosis are the fundamental pathological reactions to CAD. The primary pathological processes are cell damage and excessive cellular autophagy (5). Additionally, CAD has the further pathophysiological features of vascular dysfunction and myocardial dysfunction. Previous research has demonstrated that endothelial damage causes reduced bioavailability of nitric oxide (NO), which throws off the delicate balance between vasoconstriction and diastole. The outcomes are increased endothelial permeability and endothelial secretion disorders, further resulting in vascular dysfunction (6). Cardiovascular dysfunction is an early event of CVDs such as atherosclerosis and heart failure and can be utilized as an early predictor for the assessment of heart failure (7–9). Therefore, cardiac vascular dysfunction has become a major potential target for the treatment of heart disease.

The cardiac microvascular endothelial cell (CMEC) is the first line of defense for tissues and organs and can be used to detect alterations in hemodynamic and blood propagation signals. Together with smooth muscle cells, it regulates blood flow and plays a critical role in regulating angiogenesis and inflammatory reactions (10). The primary cause of coronary microvascular dysfunction (CMD) is endothelial dysfunction occurring in the CMECs (11), and it leads to severe tissue damage. Damaged vascular endothelial cells can have an impact on other cells and aggravate the illness (12). Moreover, the damage to CMECs precedes cardiac myocytes in the development of CVDs (13). In the event of hypoxic or inflammatory injury, among others, CMEC not only regulates nutrient exchange and oxygen-carbon cycling in cardiomyocytes but also protects cardiomyocytes from damage (14). In the heart, multiple capillary-based CMECs tightly encircle the cardiomyocytes to create a perfusion unit. The space between the two cells is extremely narrow and contiguous. For example, NO has an important regulatory role in the cardiovascular system. Decreased NO activity can lead to increased coronary artery constriction and inflammation. Two NO-converting enzymes, constitutive NO synthase (cNOS; type III) and inducible NO synthase (iNOS, type II), are highly expressed in endothelial cells (15). The released NO affects cardiac diastolic function by raising cyclic guanosine monophosphate (cGMP) levels in cardiomyocytes. However, excessive NO can induce myocardial disorders under pathological conditions (16). Several studies have demonstrated that the crosstalk between CMECs and other cells plays a crucial role in cardiac function (17, 18). Together, targeted CMECs have become a significant prospective therapeutic pathway in the management of CVD.

## 2 Composition and action mode of miRNA

MiRNAs are a class of noncoding single-strand RNA molecules typically 21–23 nucleotides in length that are encoded by endogenous genes. They have been found to play a regulatory role in gene expression (19). qPCR, *in situ* hybridization, microarrays, and RNA sequencing are some of the current techniques for miRNA detection (20). Typically, the sequence of miRNA is located within an intron or exon of a noncoding RNA (21). Most genes are transcribed by Pol II to pri-miRNA (22). The pri-miRNA is cleaved in the nucleus by Drosha and DiGeorge syndrome critical region gene 8 (DGCR8) into a pre-miRNA of approximately 70 nucleotides long (23). The pre-miRNA is transported into the cytoplasm via XPO5 and ras-associated nuclear protein (RAN), which is cleaved by Dicer into multiple double-strand miRNAs (24). After the original structure is restored in the Argonaute protein, single-strand mature miRNA is generated by eliminating the passenger strand of the miRNA double-strand body (22).

The mode of action of miRNAs is usually through the base pairing of several nucleotide sites of the miRNA with the untranslated region of the target mRNA, which in turn inhibits the expression of the target mRNA. Particularly crucial to the suppression of mRNA expression are nucleotide 2–8 sites (25). Target prediction often relies on the nucleotides in this region of the binding site (25, 26).

## 3 miRNAs a new therapeutical target

A single miRNA can control the expression of multiple target mRNAs, and a single mRNA can also be regulated by multiple miRNAs. Thus, miRNAs are generally considered promising for their potent and diverse therapeutic functional effects as well as their ability to affect multiple target genes (27). MiRNAs are critical regulators of organism development, cell differentiation, metabolism, and apoptosis (24). Clinical therapeutic strategies targeting miRNAs are already being implemented. For example, clinical study has shown that therapeutic measures targeting miRNAs in viral hepatitis C are efficacious and well tolerated (28). Beg et al. found that miRNA-43 mimics showed considerable potential for the treatment of patients with advanced cancer in phase I clinical studies (29).

MiRNAs can regulate necrotic apoptosis (30). Necrotizing apoptosis in cardiac myocytes in patients with heart disease is usually regulated by miRNAs, which in turn increases or decreases infarct size (31). For example, the hearts of pre-miR-223 knock-out mouse are more vulnerable to ischemia reperfusion (IR) injury. MiR-873 can reduce the infarct size of heart in mice with coronary artery occlusion and reperfusion by inhibiting the necrotic death of cardiomyocytes (31, 32). Although few clinical trials focus on miRNAs for CVDs, changes in the levels of particular miRNAs can often be used in the clinic as a marker to evaluate disease severity. MiRNA-133a/b, a myocardial-specific miRNA, maybe a potential target for the therapy of ventricular fibrillation after MI. Several targets associated with miRNA-133 are related to cardiac disease (33). Reduced miRNA-133a/b expression in patients is reportedly a hallmark of the development of ventricular fibrillation after MI in clinical studies (34). Additionally, miRNA-133 plays an important regulatory role in the differentiation of embryonic stem cells to cardiomyocytes (35). Patients with CAD have significantly elevated peripheral blood miRNA-218 levels, which can also be used as one of the criteria to measure the condition. This finding indicates the feasibility of monitoring variations in miRNA levels to measure and predict the progression of disease (36). Meanwhile, miRNA-133 can inhibit angiogenesis in endothelial cells by targeting vascular endothelial growth factor receptor 2 (VEGFR2) and fibroblast growth factor receptor 1 (FGFR1) (37). Cardiac microvascular injury has important implications for cardiac pathological recovery and the maintenance of physiological homeostasis (38). Therefore, this review summarizes the role and mechanism of various miRNAs in CMECs in CVDs. This review can guide the use of miRNA drugs for the clinical treatment of CVDs.

## 4 Role of miRNA in CMEC in cardiovascular disease

### 4.1 Coronary artery disease

**Figure 1:**
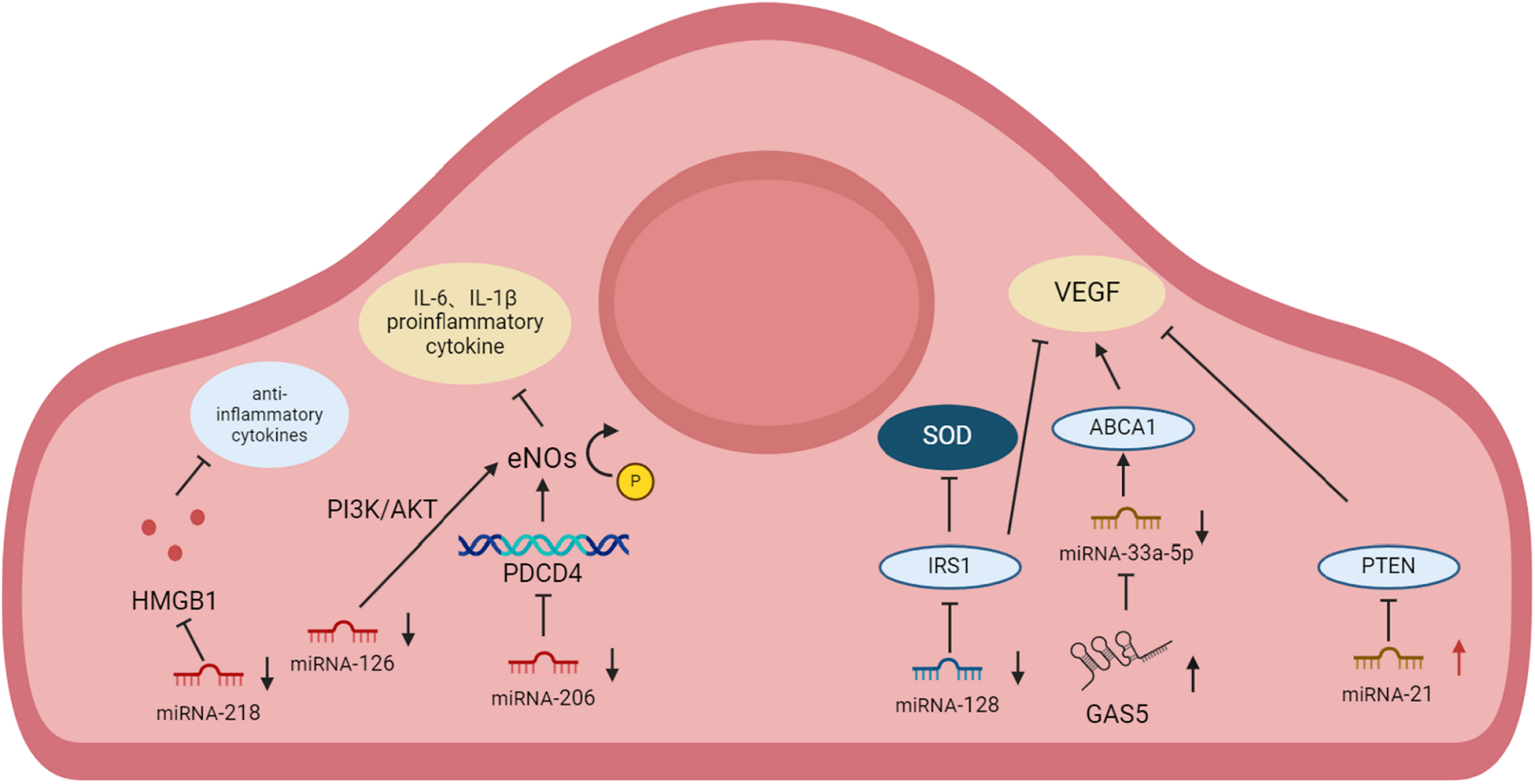
Change of expression and mechanisms of miRNA in CMEC in cardiovascular disease.

CAD is an atherosclerotic disease with inflammatory properties (3, 39). MI is a CVD caused by the formation of plaques in the interior walls of the arteries, reducing blood flow into the heart and injuring heart muscles because of insufficient oxygen supply (40). MI leads to the apoptosis of cardiac myocytes and nonmyocytes including CMECs (41), which ultimately induces apoptosis and fibrotic scarring with impaired heart function. In patients with CAD, CMD always coexist. CAD-induced reduction in perfusion pressure can trigger functional and structural alterations in microvasculature. Functional alterations include impaired vasodilator function, whereas structural alterations include arteriolar and capillary rarefaction distal to the coronary stenosis. Conversely, CMD-induced impaired vasodilatation and metabolic disturbances can exacerbate CAD (42). Previous studies have shown that CMD in the coronary vasculature is an important contributor (43, 44). The degree of improvement in the coronary microvascular system of patients with MI has a serious impact on morbidity and mortality in a clinic (45). Coronary microcirculation has become a primary target for therapies aimed at mitigating MI injury (46). The ratio of cardiac CMECs to cardiomyocytes is about 3:1, which determines its importance in cardiovascular health (17). Therefore, we summarize the roles and mechanisms of various miRNAs in CAD in CMECs and briefly describe them in Figure 1.

#### 4.1.1 miRNA-218

MiRNA-218 is located at the introns of the SLIT2 and SLIT3 genes (47, 48). In rat primary CMECs, miRNA-218 is significantly lower after homocysteine (Hcy) treatment than in the control group. Hcy-induced reductions in NO bioavailability result in impaired endothelium capacity to regulate vascular tone. Hcy-induced oxidative stress causes the oxidative inactivation of NO and endothelial NO synthase (eNOS) uncoupling and contributing to the formation of more reactive oxygen species (ROS) (49, 50). Elevated Hcy levels are considered a risk factor for the development of atherosclerotic vascular injuries in coronary heart disease (51). Therefore, a CAD model is usually built by Hcy (52). Overexpression of miRNA-218 alleviates Hcy-induced apoptosis, pro-inflammatory cytokine release, and improved CMEC migration. When CMEC injury occurs in advanced atherosclerosis, miRNA-218 expression is likewise decreased to increase high mobility group protein B1 (HMGB1) expression. HMGB1 is released from macrophages/monocytes upon stimulation with lipopolysaccharide (LPS), tumor necrosis factor (TNF), interleukin-1beta (IL-1β), and necrotic cells. HMGB1 binds to cell surface receptors and mediates the inflammatory response via the mitogen-activated protein kinase (MAPK) signal pathway. In clinical studies, miRNA-218 is also found to be significantly higher than normal in the peripheral blood of patients with CAD, which can be one of the criteria for diagnosing CAD (36, 53).

#### 4.1.2 miRNA-26b-5p

MiRNA-26b-5p is a member of the miR-26 family, which is located at CHR2 gene in human. Bidzhekov et al. have demonstrated that miRNA-26b-5p is also associated with atherosclerosis, the main cause of CVDs (54). In the MI model (55), the long non-coding RNA (lncRNA) expression of Malat1 is significantly elevated in injured CMEC after 7 days. LncRNAs are transcripts >200 nucleotides long which modulate transcription, epigenetic modifications, and posttranslational modifications by interacting with DNA and signaling receptors (56). Previous study has shown that Malat1 plays an important regulatory role by sponging miRNAs in CVD and endothelial dysfunction (57). Its specific target miRNA-26b-5p causes a significant increase in the expression of downstream signaling factor mitofusin 1 (MFN1). MFN1 is usually involved in oxidative stress pathways and mitochondrial apoptosis, such as decreasing ROS activity, maintaining mitochondrial homeostasis in CMEC thereby alleviating cardiac microvascular dysfunction. What's more, Malat1 is the competing endogenous RNA (ceRNA) for miR-26b-5p. Knockdown of Malat1 caused an exacerbation of MI, suggesting that endogenous miRNAs are also critical for the development of CVD. However, miRNA-26b-5p expression in circulating blood is significantly lower in heart failure patients than in normal subjects in clinical trials and is positively correlated with ejection fraction (58).

#### 4.1.3 miRNA-128

MiRNA-128 is embedded on CHR 2q21.3 and 3p22.3 (59). MiRNA-128 is often considered as a potential therapeutic target to regulate vascular smooth muscle cells (60). The study has shown that overexpression of miRNA-128 promotes mitochondrial dysfunction by directly targeting NADH Dehydrogenase (Ubiquinone) Fe-S Protein 4 (NDUFS4) (61). MiRNA-128 in CMEC targets IRS1 in the Hcy-induced coronary heart disease model (62). Inhibition of IRS1 expression promotes vascular endothelial growth factor (VEGF) expression inhibits oxidative stress, and enhances the anti-apoptotic capacity and migration ability of CMEC.

#### 4.1.4 miRNA-33a-5p

MiRNA-33a-5p is an intron miRNA that is located inside the intron sequence of the sterol-response-element-binding protein gene 2 (SREBP2) (63). The study has shown that miR-33a-5p promotes apoptosis via targeting nodal modulator1 (NOMO1) and other targets (64). After treatment with Hcy in the CMEC vascular dysfunction model, growth-arrest specific transcript 5 (GAS5) expression is markedly reduced whereas miRNA-33a-5p expression is elevated (65). GAS5 would compete with miRNA-33a-5p to bind the target ATP-binding cassette transporter A1 (ABCA1), while miRNA-33a-5p action on ABCA1 would promote apoptosis and inhibit cell growth (66).

#### 4.1.5 miRNA-21

MiRNA-21 is located on CHR 17 in human. The study has shown that miRNA-21 enhances hypoxia inducible factor-1α (HIF-1α) activity via targeting Hsc70-interacting protein (CHIP), playing a role in promoting angiogenesis (67). In a rat MI model simulated by left anterior descending branch ligation (LAD) infarct surgery, miRNA-21 expression was elevated in CMEC. MiRNA-21 has an impact on the downstream target phosphatase and tensin homolog deleted on phosphatase and tensin homolog (PTEN), regulates protein kinase B (AKT) and extracellular regulated kinases (ERK) pathway, promotes VEGF expression, which ameliorates the injury caused by acute infarction. And when miRNA-21 inhibitors are administered, angiogenesis is inhibited to aggravate the disease (68, 69).

#### 4.1.6 Other miRNAs

LncRNA HULC expression is downregulated in cardiac tissue in a LAD infarct model. HULC can interact directly with miRNA-29b to inhibit inflammatory cytokines such as interleukin-6 (IL-6) and interleukin-8 (IL-8) and promote angiogenesis. And miRNA-29b overexpression in CMEC eliminates the effect of HULC and exacerbates the inflammatory response (70). In primary CMEC isolated from rats with MI, the level of miRNA-223-3p is considerably increased during the migratory and proliferative phases (71), which can directly target the gene recombinant ribosomal protein S6 kinase Beta 1 (RPS6KB1) and participate in the RPS6KB1/HIF-1α signaling pathway. This results in the cell being blocked in the S-phase of the cell cycle, inhibiting migration and proliferation of ischemic CMEC.

### 4.2 Ischemia reperfusion

Reperfusion is currently the only effective strategy to address cardiac ischemia (72). Thrombolysis, percutaneous coronary intervention, and coronary artery bypass surgery have been applied in clinics (72, 73). However, these techniques inflict secondary damage due to myocardial IR injury (74). IR injury not only leads to necrotic apoptosis and autophagy due to excessive ROS release (75, 76), but also activates senescent cells and exacerbates fibrosis formation (77, 78). Previous studies have shown that endothelial function plays a fundamental role in the protection against IR injury (79). IR is a major cause of the coronary microcirculation injury, inducing endothelial cell dysfunction and increased vascular leakage. Conversely, ischemia alone has a limited effect (80). Thus, understanding the functional role of miRNAs under IR injury is crucial.

#### 4.2.1 miRNA-206

MiRNA-206 is a member of the miRNA-1 family, which is located between the interleukin-17 gene and the polycystic kidney and hepatic disease 1 gene in human (chr 6), mouse (chr 1) and rat (chr 9) (81). MiRNA-206 normally regulates the function of skeletal muscle. Once its dysregulation is usually accompanied by skeletal muscle disorders such as duchenne muscular dystrophy (DMD) and amyotrophic lateral sclerosis (ALS) (82). In the hypoxia/reoxygenation (H/R) model, YAP expression is reduced and miRNA-206 expression is downregulated within CMEC. Elevated expression of its direct target gene, programmed cell death 4 (PDCD4), decreases eNOS phosphorylation and inhibits platelet endothelial cell adhesion molecule-1 (PECAM-1) expression with CMEC damage (83). PDCD4 may improve the mitigation of inflammatory responses as well as oxidative stress through the heme oxygenase 1 (HO-1) pathway (84).

#### 4.2.2 miRNA-126

MiRNA-126 is located on CHR 9 in human, which is involved in cell apoptosis, inflammation, proliferation, angiogenesis, and other processes by negatively targeting phosphatidylinositol 3-kinase (PI3K), VEGF, vascular cell adhesion molecule-1 (VCAM-1), and low density lipoprotein receptor related protein (LRP6) (85). Yang et al. found that miRNA-126 expression is reduced in CMEC after H/R treatment. MiRNA-126 reduced pro-inflammatory cytokine expression and controlled the inflammatory response by stimulating the PI3K/Akt/eNOS signaling pathway in human CMEC. When miRNA-126 expression is reduced, the anti-inflammatory effects and lumen formation are inhibited after CMEC injury (86).

#### 4.2.3 miRNA-200a

MiRNA-200a is located on CHR 1p36.33 (87). The study has shown that miRNA-200a is highly sensitive to ROS (88). In the H/R model, the level of ROS increases in both cardiomyocytes and CMEC, and miRNA-200a expression reduced in CMEC. Accordingly, miRNA-200a is usually involved in pathway responses related to oxidative stress. When miRNA-200a is overexpressed in H/R-treated CMEC, apoptosis is ameliorated. The mechanism may be through inhibiting the expression of oxidative stress pathways such as nuclear factor erythroid 2-related factor 2 (NRF2) (89, 90).

#### 4.2.4 Other miRNAs

In the IR model, miRNA-495 expression is decreased, while pro-inflammatory cytokine expression is increased in CMEC. Excessive miRNA-495 can inhibit pyrin domain-containing 3 (NLRP3), thereby reducing the secretion of pro-inflammatory cytokines such as IL-6 and TNF-α. It alleviates inflammation while enhancing CMEC cell activity as well as migration ability (91). MiRNA-145-5p levels decreases in patients with coronary artery disease, while its overexpression effectively alleviates H/R-induced CMEC injury by inhibiting mothers against decapentaplegic homolog 4 (SMAD4). SMAD4 is a crucial element of the transforming growth factor β (TGFβ) signaling pathway and is involved in cell proliferation, differentiation, migration, and apoptosis (92).

### 4.3 Heart failure

Heart failure is the end stage in the development of various heart diseases, such as MI and myocarditis. Several studies show that endothelium-dependent vasodilation is blunted in patients with heart failure (8, 93, 94). Currently, Nebivolol is often used clinically to treat heart failure. Nebivolol plays a role in the beta (3)-adrenoceptor agonistic effect on endothelial cells that stimulates eNOS, as well as in repairing vascular dysfunction (95, 96). Nebivolol also inhibits the high salt-induced elevated expression of miRNA-320, which is located in CMECs and is involved in IR injury after MI via the antithetical regulation of heat-shock protein 20 (Hsp20) (97–99). Thus, improving vascular dysfunction has become an important direction in the treatment of heart failure in clinics.

#### 4.3.1 miRNA-1-3p

MiR-1-3p is encoded by the miR-1-2 gene located on CHR 18q11.2 (100). Wang et al. found that the level of miRNA-1-3p is decreased in CMEC when chronic heart failure occurs (101). When a vast array of miRNA-1-3p is secreted, it could directly inhibit the expression of endothelin-1 (ET-1), improve vascular endothelial dysfunction and cardiac remodeling. Moreover, up-regulated miR-1-3p inhibits cell proliferation and promotes apoptosis by targeting stress-associated endoplasmic reticulum protein 1 (SERP1), which leads to dysfunction of CMECs (102). We summarize the miRNA of CMEC in cardiovascular disease to provide a theoretical basis (Table 1, Figure 1).

**Table 1:** miRNAs with altered expression in CMEC in cardiovascular disease.

miRNA	Subject of experiment	Disease	Changes of expression	Target	Function	Reference
miRNA-218	CMEC of Sprague-Dawley (SD) rats	Atherosclerosis	↓	HMGB1	Inhibits HMGB1 interacting with a large number of anti-inflammatory factors	(36, 53)
miRNA-33a-5p	CMEC of SD rats	Atherosclerosis	↑	ABCA1	Promotes apoptosis	(65, 66)
miRNA-128	CMEC of SD rats	Coronary heart disease	↓	IRS1	Promotes VEGF expression and increases SOD activity	(62)
miR-26b-5p	CMEC of C57BL/6 mice	MI	↓ (7 days)	MFN1	Inhibits MFN1 expression and elevates ROS activity	(55)
miRNA-21	CMEC of SD rats	MI	↑	PTEN	Promotes VEGF expression	(68, 69)
miRNA-29b	CMEC of SD rats	MI	↑		Promotes the expression of inflammatory cytokines such as IL-6 and IL-8	(70)
miRNA-223-3p	CMEC of SD rats	MI	↓	RPS6KB1	Participates in the RPS6KB1/HIF-1α signaling pathway and promotes angiogenesis	(71)
miRNA-206	CMEC of Wistar rats	IR	↓	PDCD4	Enhances eNOS phosphorylation and promotes PECAM-1 expression	(83, 84)
miRNA-126	Human cardiac microvascular endothelial cell (HCMEC)	IR	↓	PI3K	Activates PI3K/Akt/eNOS signaling pathway and exerts anti-inflammatory effects	(86)
miRNA-200a	Human coronary endothelial cell (HCAEC)	IR	↓	NRF2	Inhibits the expression of oxidative stress pathways such as NRF2	(89, 90)
miRNA-495	CMEC of C57BL/6 mice	IR	↓	NLRP3	Inhibits pro-inflammatory cytokines	(91)
miRNA-145-5p	HCMEC	IR	↓	SMAD4	Inhibits SMAD4 and inhibits cell proliferation and migration	(92)
miRNA-1-3p	Rat cardiac microvascular endothelial cell (RCMEC)	Heart failure	↓	ET-1	Inhibits target ET-1 expression and improves vascular endothelial dysfunction	(101)

## 5 Role of miRNA in CMECs in diabetes

Diabetes mellitus (DM), caused either by deficient insulin secretion or damaged of pancreatic β cell consists of type I and type II DM. Type I DM is an autoimmune disease that affects pancreatic cells and reduces insulin production. Type II DM is a disease caused by the dysfunction or defect of pancreatic β cells, which reduces insulin signaling and secretion from the pancreas (103). Diabetic patients have elevated blood glucose due to islet damage. High intracellular glucose levels usually lead to metabolic abnormalities with a variety of complications, including cardiac microangiopathy (104). Endothelial cells are some of the first cell types to be exposed to hyperglycemia (105). Hyperglycaemia-induced mitochondrial dysfunction and endoplasmic reticulum stress are associated with mechanisms, such as the activation of protein kinase C (PKC), polyol, and hexosamine pathways. Hyperglycaemic lesion also induces ROS accumulation that damages proteins or DNA and modulates intracellular signaling pathways, such as mitogen activated protein kinases and redox sensitive transcription factors (106). In hyperglycemic CMECs, the glycolytic reserve is significantly reduced, and the ability to produce ATP via glycolysis is impaired (107). Additionally, higher ROS levels and oxidative stress brought on by hyperglycemia frequently result in endothelial cell apoptosis, lowering the mRNA levels of oxidative phosphorylation (OXPHOS)-related genes. Thus, diabetes-induced hyperglycemia damages CMECs through oxidative stress, as well as inhibits mitochondrial glycolysis and the ability of OXPHOS to generate ATP. In turn, CMEC proliferation is inhibited, apoptosis is promoted (107–109). Moreover, this damaged CMECs activate PKC, and release inflammatory cytokines and adhesion factors (110, 111).

Clinical research is gradually focusing on targeting miRNAs in the blood of diabetic patients. Studies have shown that diabetic patients with high plasma miRNA-19a expression have less vascular circulating tissue factor (TF) expression (112). TF is a procoagulant factor released by vascular endothelial cells, which are suppressed by miRNA-19a and miRNA-126. TF content in the peripheral blood of type II diabetic patients is also significantly negatively correlated with miRNA-181b (113). We summarize the miRNA of CMEC in diabetes (Table 2).

**Table 2:** miRNAs with altered expression in CMEC in diabetes.

miRNA	Subject of experiment	Changes of expression	Target	Function	Reference
miRNA-92a	Mice cardiac microvascular endothelial cell (MCMEC) and HCMEC	↑	ADAM10, KLF2, KLF4	Inhibits ADAM10, KLF2 and KLF4 expression and inhibits cell migration	(114)
miRNA-503	Human microvascular endothelial cell line (HMEC-1)	↑	Apelin-12	Promotes pro-inflammatory cytokine release through the JNK and p38MAPK signaling pathways	(115)
miRNA-216b	RCMEC	↑	FZD5	Inhibits CMEC proliferation capacity and migration ability	(116)
miRNA-193-5p	CMEC of Wistar rats	↑	IGF2	Inhibits cell proliferation	(117)
miRNA-200b	MCMEC	↓	VEGF, p300, ZEB1, ZEB2	Reduces expression of endothelial-specific markers such as VEGF and CD31	(118)
miRNA-146a	MCMEC and HCMEC	↓	TRAF6, IRAK1	Promotes the release of pro-inflammatory cytokines such as IL-6 and IL-1β	(119)

### 5.1 miRNA-92a

Mature miRNA-92a can be produced by miR-92a-1 and miR-92a-2 manipulation. MiRNA-92a-1 is situated within the third intron of an open reading frame 25 (C13orf25) gene located at chromosome 13q31-q32 (120). MiRNA-92a-2 is encoded in miR-106-363 cluster on the X chromosome, region q26.2 (121), which is of great importance in cardiac development and angiogenesis. According to the study, high glucose induction dramatically increases the level of miRNA-92a in human CMECs, reduces the ability of cells to migrate and proliferate, and increases inflammatory response. MiRNA-92a can inhibit the expression of A disintegrin and metalloproteinase 10 (ADAM10), Krüpple-like factor2 (KLF2) and Krüpple-like factor4 (KLF4). ADAM10 promotes cell migration, KLF2 and KLF4 are important transcriptional regulators of anti-inflammatory and antioxidant activity (114). Therefore, cells in the high glucose group with elevated miRNA-92a expression have a significantly reduced ability to migrate and proliferate, and an increased inflammatory response.

### 5.2 miRNA-146a

MiRNA-146a is located on CHR 5, which is an important element in the regulation of inflammatory responses (122). Previous study has shown that miRNA-146a inhibits the target gene TGF-β1 to anti-angiogenesis. The high glucose treatment of CMECs induces a decrease in miRNA-146a expression with increasing the expression of tumor necrosis factor receptor associated factor 6 (TRAF6) and interleukin-1 Receptor-Associated Kinase 1 (IRAK1) (119). Promoting the release of pro-inflammatory cytokines, such as IL-6 and IL-1β, increases inflammatory response and promotes fibrosis. Moreover, when normal CMEC's miRNA-146a expression is suppressed, it also causes hyperglycemic lesions.

### 5.3 miRNA-503

MiRNA-503 is located on chromosome Xq26.3 and is elevated in the high-glucose treated CMEC model (115, 123). MiRNA-503 inhibits Apelin-12 levels by binding to the promoter region of Apelin gene, which can promote the release of pro-inflammatory cytokines such as TNF-α, IL-6, and IL-1β through c-Jun N-terminal kinase (JNK) and p38 MAPK signaling pathways, further causing vascular dysfunction and the development of heart failure in severe cases (115).

### 5.4 Other miRNAs

Elevated miR-216b expression in diabetic CMECs inhibits the expression of its downstream target frizzled class receptor 5 (FZD5), while decreased miR-216b level inhibits the proliferative capacity and migratory capacity of CMEC (116). In addition, miR-193-5p expression is increased in CMECs, which interacts with the direct target insulin growth factor 2 (IGF2) to inhibit cell proliferation (117). Feng et al. show that hyperglycemia in diabetic models is expressed by suppressing endothelial cell miRNA-200b (118). MiRNA-200b prevents endothelial mesenchymal transition (EndMT) which is caused by decreased levels of endothelial-specific markers like VEGF and PECAM-1, which impact greatly on heart failure and cardiac fibrosis.

## 6 miRNAs in cell crosstalk

**Figure 2:**
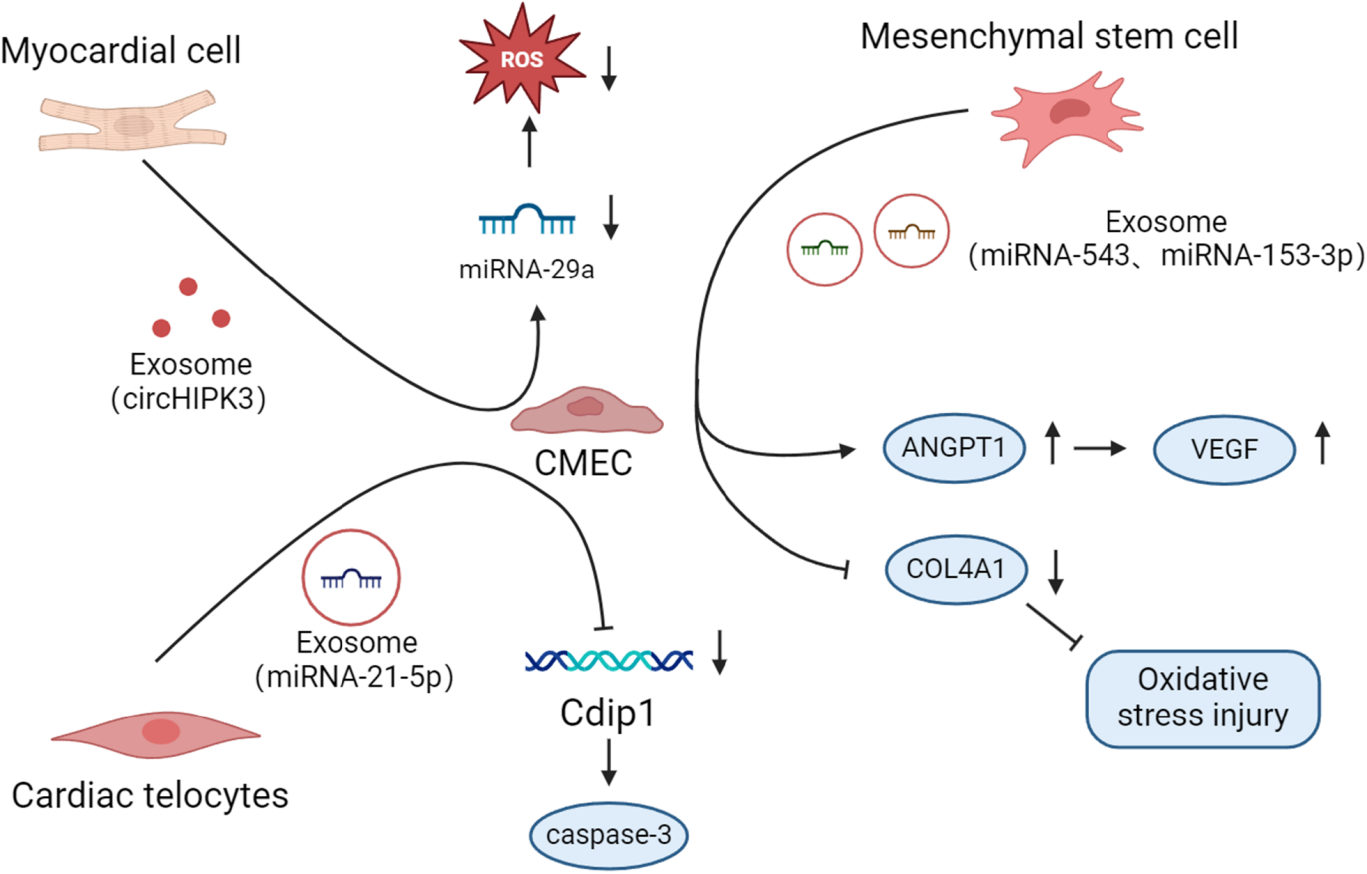
Mechanisms by which CMEC crosstalk with other cells occurs in disease.

MiRNAs play an important role in cellular crosstalk, especially in cardiovascular aspects. We summarize the crosstalk mechanisms between other cells in the heart and CMEC (Figure 2).

### 6.1 Cardiomyocytes

One cardiomyocyte is typically surrounded by multiple CMECs in the heart to create a perfusion unit, and the space between the two cells is incredibly narrow. Consequently, the interaction between CMECs and cardiomyocytes has significant effects on heart function (18).

Hypoxia-treated cardiomyocytes release exosomes containing excessive circHIPK3, which acts as an endogenous miR-29a sponge. Exosomes then enter oxidative stress-injured CMECs to inhibit miRNA-29a activity, drive increased insulin-like growth factor 1 (IGF-1) expression, and reduce ROS and malondialdehyde (MDA) expression (124). In contrast to the above results (125), in the H/R model, injured cardiomyocytes release a large amount of miRNA-499. Subsequently, miRNA-499 translates into CMEC to inhibit the level of α7-nachr, promote an inflammatory response, and exacerbate vascular dysfunction.

CMEC also affects cardiomyocytes. Hypoxia-treated primary CMECs *in vitro* release miRNA-27b-3p-rich exosomes to prevent cardiomyocyte damage (126). MiRNA-27b-3p-rich exosomes released from hypoxia-treated CMECs injected in advance into the myocardium significantly inhibit H/R-induced MI injury in rats. The mechanism involves the ability of miRNA-27b-3p to significantly inhibit Foxo1 expression and this suppress cardiomyocyte apoptosis. Moreover, rat hypoxia-treated CMECs release miRNA-210-3p-rich exosomes that are delivered to H/R-treated cardiomyocytes. MiRNA-210-3p further targets transferrin receptor (TFR) in cardiomyocytes to alleviate cardiomyocyte ferroptosis (127).

### 6.2 Cardiac telocytes

Cardiac telocyte (CT) is a population of cells with a very small gap to cardiac muscle cells (128). When a cardiac infarction occurs, CTs in the area of ischemic infarction die in large numbers. Conversely, CT cell density is gradually restored during infarct healing. When CT is therapeutically transplanted into the damaged myocardial area, it can effectively improve myocardial function and the degree of myocardial fibrosis (129). Additionally, CT can release exosomes and transfer them to hypoxic CMECs, and the most abundant miRNA in the exosomes is miRNA-21-5p (130). MiRNA-21-5p enters CMECs to repress the target gene cell death inducing p53 target 1 (Cdip1), thereby downregulating activated caspase-3 and inhibiting apoptosis.

### 6.3 Mesenchymal stem cells

Mesenchymal stem cells (MSCs) are a group of multipotent precursor cells that can differentiate into multiple cell types and are used clinically to address hematologic and CVDs. MSC can secrete immunomodulatory and angiogenic bioactive factors that promote tissue repair and regeneration (131, 132). Previous studies have shown that MSC is beginning to become the focus of clinical studies for the treatment of AMI and ischemic heart failure (133). Moreover, hMSC can out miR-543-rich exosomes that transfer into hypoxia-treated CMEC to down-regulate recombinant collagen type IV alpha 1 (COL4A1) expression and ameliorate hypoxic injury (134). Ning et al. showed that MSCs protected cardiac endothelial cells from damage by releasing miRNA-153-3p low-expressing exosomes and activating the Angiopoietin-1 (ANGPT1) and VEGF/VEGFR2/PI3K/Akt/eNOS pathways (135).

## 7 Discussion

A single miRNA can have multitargeting effects, so miRNA-targeted therapeutic approaches have recently become a research hotspot with great prospects. For example, miRNA-145 can reduce in-stent restenosis rate via anti-vascular proliferation with no side effects (136). Improving microvascular endothelial dysfunction has also become an important direction for studying CVD treatment. This review summarizes the roles played by numerous significant miRNAs in CMECs and their mechanisms of action, with the aim of providing a theoretical basis for the clinical use of miRNA therapies. This review also elaborates on the role of miRNAs in the crosstalk between CMECs and other cells in the heart and summarizes the mechanisms of action. MiRNAs in diseases are currently being investigated further and are frequently used as diagnostic criteria (36). However, some remaining issues with miRNA therapy need to be resolved, including the requirement for extremely accurate miRNA sequence prediction and a certain number of miRNAs with highly conserved sequences among different organisms. MiRNA-targeted treatment will undoubtedly advance with the improvement in detecting technologies, such as gene sequencing. Furthermore, miRNA therapy has the advantage of multitargeting, which theoretically fits cardiovascular therapies well. This promising future will undoubtedly serve as a catalyst for the development of cardiovascular therapies.
